# Neural mechanisms underlying the effects of face-based affective signals on memory for faces: a tentative model

**DOI:** 10.3389/fnint.2012.00050

**Published:** 2012-07-24

**Authors:** Takashi Tsukiura

**Affiliations:** Department of Cognitive and Behavioral Sciences, Graduate School of Human and Environmental Studies, Kyoto UniversityKyoto, Japan

**Keywords:** fMRI, face, memory, amygdala, orbitofrontal cortex, insula

## Abstract

In our daily lives, we form some impressions of other people. Although those impressions are affected by many factors, face-based affective signals such as facial expression, facial attractiveness, or trustworthiness are important. Previous psychological studies have demonstrated the impact of facial impressions on remembering other people, but little is known about the neural mechanisms underlying this psychological process. The purpose of this article is to review recent functional MRI (fMRI) studies to investigate the effects of face-based affective signals including facial expression, facial attractiveness, and trustworthiness on memory for faces, and to propose a tentative concept for understanding this affective-cognitive interaction. On the basis of the aforementioned research, three brain regions are potentially involved in the processing of face-based affective signals. The first candidate is the amygdala, where activity is generally modulated by both affectively positive and negative signals from faces. Activity in the orbitofrontal cortex (OFC), as the second candidate, increases as a function of perceived positive signals from faces; whereas activity in the insular cortex, as the third candidate, reflects a function of face-based negative signals. In addition, neuroscientific studies have reported that the three regions are functionally connected to the memory-related hippocampal regions. These findings suggest that the effects of face-based affective signals on memory for faces could be modulated by interactions between the regions associated with the processing of face-based affective signals and the hippocampus as a memory-related region.

## Introduction

Facial stimuli convey various types of information in human society, and are very important in non-verbal communication with others. For example, when we encounter someone for the first time, we try to read their feelings from their face. The facial information that can provide us with impressions of people includes many factors such as trustworthy, caring, responsible, emotionally stable, sociable, attractive, intelligent, confident, dominant, happy, aggressive threatening, mean, or weird features (Oosterhof and Todorov, 2008; Todorov and Engell, 2008; Todorov et al., 2008b), and it is also influenced by racial information (Stanley et al., 2011, 2012). Among them, three possible factors of facial expression, attractiveness, or trustworthiness are particularly important in forming the impressions of people and in selecting which people should be remembered, because the response times for the attractiveness and trustworthiness judgments were almost identical and faster than the response times for judgments of competence, likeability, and aggressiveness (Willis and Todorov, 2006). However, there is little evidence of the neural mechanisms underlying this psychological process. The aims of this article are to review psychological and cognitive neuroscience studies related to the effects of face-based affective signals on memory for faces, particularly those concerning facial expressions, attractiveness, and trustworthiness, and to propose a tentative framework for understanding the neural mechanisms of memory for faces in the context of human social interaction.

## Effects of face-based affective signals on memory for faces: psychological studies

The first main factor of face-based affective signals is facial expression. Previous psychological studies have reported that positive facial expressions such as smiling have a beneficial effect on remembering faces (D'Argembeau et al., 2003; D'Argembeau and van der Linden, 2004, 2007; Laroi et al., 2006; Shimamura et al., 2006; Ebner and Johnson, 2009). For example, Shimamura and his colleagues found that faces encoded with smiling expressions were remembered more accurately than those with other expressions, including surprise, anger, or fear (Shimamura et al., 2006). However, there is also evidence from psychological studies that positive facial expressions have no selective advantage on memory for faces. One study showed better remembering of faces with fearful expressions than of those with happy expressions (Righi et al., 2012), whereas another study reported memory enhancement for faces with positive and negative expressions, compared to those with neutral expressions (Foa et al., 2000). Taken together, both positive and negative facial expressions could contribute to the enhancing effects on memory for faces.

The second main factor of affective signals conveyed from faces is facial attractiveness. The beneficial power of facial attractiveness has been observed in several studies investigating the recognition of other people. For example, adults (Langlois et al., 2000) as well as infants (Langlois et al., 1987) show a preference for attractive faces. Also, compared to facially unattractive people, facially attractive people are likely to show enhanced positive behavior in a social way (Langlois et al., 2000), and to be judged people with better personality by others (Dion et al., 1972). The positive bias toward attractive faces has been identified in memory for faces, in which attractive faces are better remembered than unattractive faces (Cross et al., 1971; Marzi and Viggiano, 2010). However, there is evidence from psychological studies that the distinctiveness in attractive faces is low, and the low distinctiveness of faces has little beneficial effect on memory for faces (Light et al., 1981). Thus, the effect of facial attractiveness on memory for faces may be advantageous, but the mechanisms underlying it and its efficiency in terms of remembering faces are still controversial.

The third main factor of face-based affective signals enhancing the memory processing of faces is facial trustworthiness. There is evidence from psychological studies that facial untrustworthiness has a beneficial power on memory for faces. For example, one psychological study demonstrated that the recognition of faces with untrustworthy impressions was better than that of faces with neutral or trustworthy impressions (Mealey et al., 1996). Another psychological study found that the faces of trustworthy-looking people with bad personality traits were remembered more accurately than those of untrustworthy-looking people with bad personality traits (Suzuki and Suga, 2010). In this study, 58 college students played a debt game, where they learned to discriminate among good, neutral, and bad lenders, who respectively charged no, moderate, and high interest on the debt. Each lender had either a trustworthy- or untrustworthy-looking faces. The findings of better memory for people associated with an untrustworthy impression or bad personality, which depends on the face-based first impression or on the later contextual information of personality, suggest that humans could be equipped with protective mechanisms against people with really bad personality traits (Suzuki and Suga, 2010).

## Roles of the amygdala in the processing of face-based affective signals

**Figure 1:**
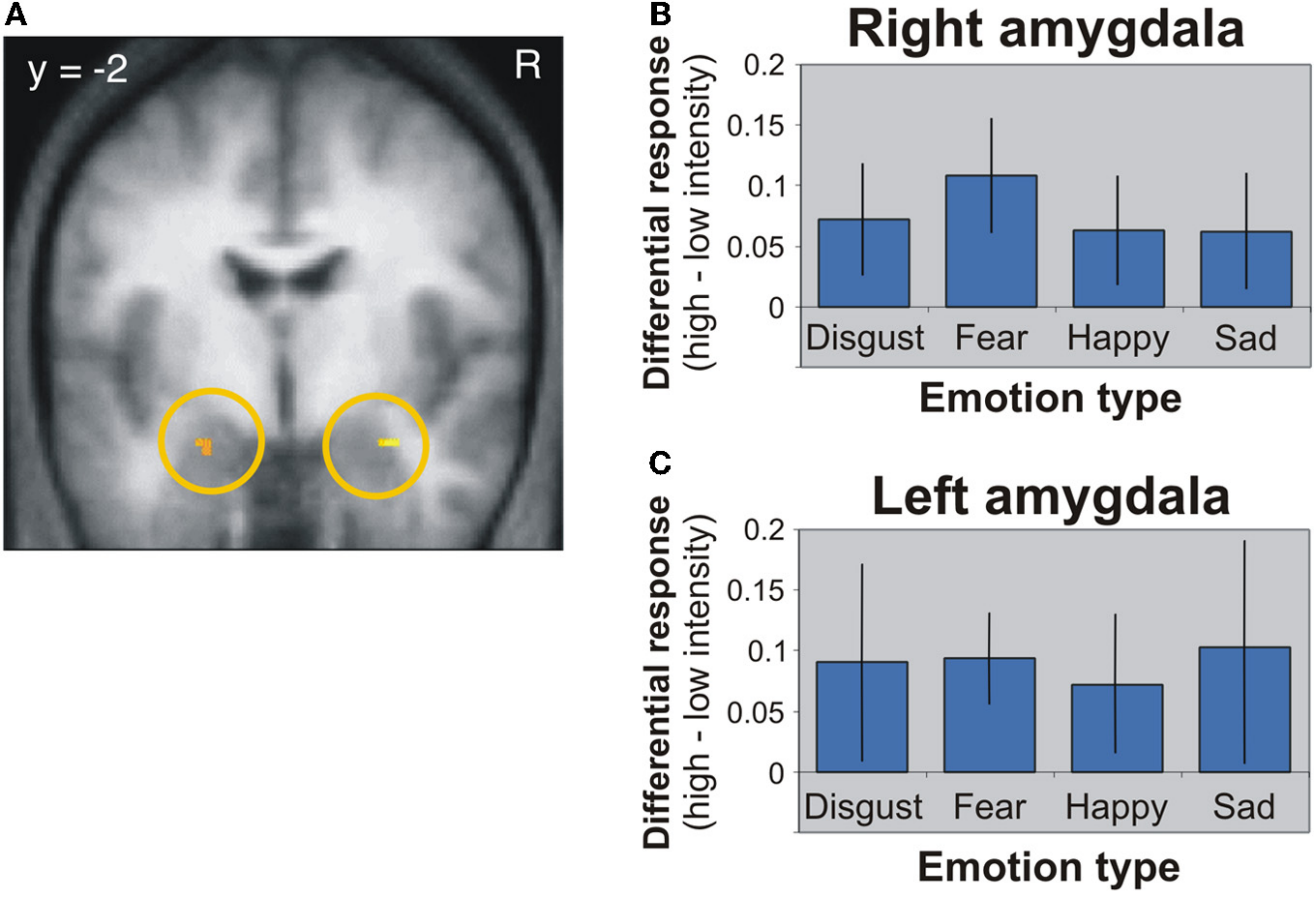
**Amygdala activation in response to different facial expressions (Winston et al., 2003). (A)** Activation image of the amygdala during the processing of high-intensity expressions compared to low-intensity expressions. **(B)** Activation profiles of the right amygdala. **(C)** Activation profiles of the left amygdala. This figure was reused with the permission.

The first candidate region associated with the processing of face-based affective signals is the amygdala (Figure 1). Previous functional neuroimaging studies have reported that the amygdala shows significant activations during the processing of face-based negative and positive signals including happy, sad, and fearful expressions (Fusar-Poli et al., 2009). For example, activity of the amygdala was greater during perceiving high-intensity expressions than low-intensity expressions, and the activity was identified in relation to both positive and negative signals from faces (Winston et al., 2003). This suggests that activity of the amygdala could be modulated by affective intensities of facial expressions. However, there is functional neuroimaging evidence linking amygdala activities to the processing of negative facial expressions (Vuilleumier and Pourtois, 2007). For example, one functional MRI (fMRI) study demonstrated that the amygdala showed greater activity during the processing of negative facial expressions than of neutral facial expressions (Iidaka et al., 2001). Amygdala responses selective to negative facial expressions have been observed in other functional neuroimaging and lesion studies (Adolphs et al., 1994; Morris et al., 1996; Broks et al., 1998). The amygdala, in which the activity is modulated by affective intensities of facial expressions, could be involved in the processing of both positive and negative facial expressions, but the involvement could be biased toward more negative expressions. However, given that a meta-analysis study showed no effect of angry and disgusted expressions on amygdala activations (Fusar-Poli et al., 2009), further investigations would be required to clarify whether the amygdala activations are modulated only by specific types of facial expressions, rather than affective intensities of facial expressions.

The contribution of the amygdala to the processing of facial attractiveness or trustworthiness has also been demonstrated in cognitive neuroscience studies (Bzdok et al., 2011). Several functional neuroimaging studies have reported greater amygdala activity in the processing of highly attractive and unattractive faces than of middle-ranked attractive faces (Winston et al., 2007; Cloutier et al., 2008; Liang et al., 2010). Additionally, there is functional neuroimaging evidence that amygdala activity increased during the processing of both trustworthy and untrustworthy faces, compared to that of neutral faces (Todorov, 2008). For example, one fMRI study found that the amygdala showed greater responses to highly trustworthy as well as to highly untrustworthy faces than to neutral faces (Said et al., 2009). The non-linear amygdala responses to face-based affective signals suggest that this region could be involved in sensing the value of social stimuli including both positive and negative affects of faces. However, other functional neuroimaging studies have demonstrated that the amygdala response to faces increased as their perceived untrustworthiness increased (Winston et al., 2002; Engell et al., 2007; Todorov et al., 2008a), and this amygdala response to untrustworthy faces was supported by a lesion study (Adolphs et al., 1998). Taken together, amygdala responses to facial attractiveness and trustworthiness could be possibly modulated by both good and bad impressions of the two factors, but the responses could be biased to more negative signals conveyed from faces. However, further studies would be required to clarify whether the amygdala activity is modulated by facial impressions or by affective intensities of presented pictures.

## Roles of the orbitofrontal cortex in the processing of face-based affective signals

**Figure 2:**
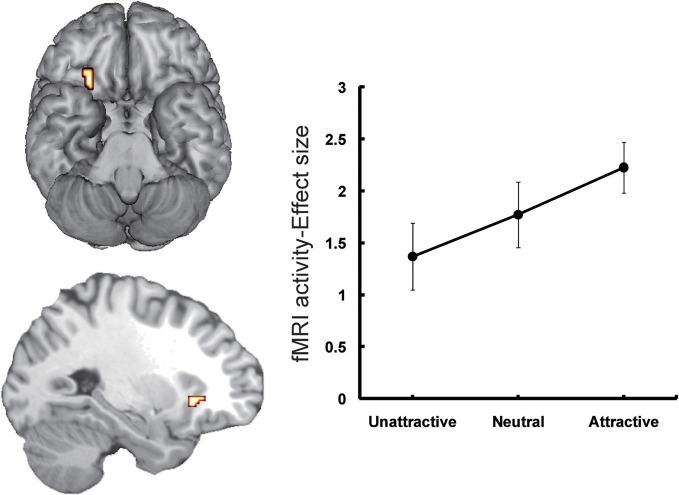
**Orbitofrontal activations in response to attractive faces (Tsukiura and Cabeza, 2011a).** Activity of the OFC reflected an increasing function of facial attractiveness. This figure was reused with the permission.

The second candidate region associated with the processing of face-based affective signals is the medial orbitofrontal cortex (OFC: Figure 2). The involvement of the medial OFC region in the processing of facial expressions has been identified when the facial expression is happy or smiling. For example, activity of the medial OFC region was enhanced in the processing of happy facial expressions, compared to that of facial expressions of disgust (Gorno-Tempini et al., 2001). The medial OFC responses to happy facial expressions have also been found in other functional neuroimaging studies (O'Doherty et al., 2003; Minagawa-Kawai et al., 2009). However, given that a meta-analysis study failed to identify significant activations in the medial OFC region during the processing of happy facial expressions (Fusar-Poli et al., 2009), it is possible that a greater response of this region to happy facial expressions may be limited in some specific situations, such as the explicit processing of happy facial expressions (Gorno-Tempini et al., 2001) or the social processing of smiling faces (Minagawa-Kawai et al., 2009).

Moreover, functional neuroimaging studies have linked the medial OFC regions to the processing of attractive faces as one of the positive affective signals from faces (Aharon et al., 2001; O'Doherty et al., 2003; Kranz and Ishai, 2006; Bray and O'Doherty, 2007; Ishai, 2007; Winston et al., 2007; Cloutier et al., 2008; Liang et al., 2010; Tsukiura and Cabeza, 2011a,b). The roles of this region in the processing of face-based positive signals have also been observed when the personality traits estimated from faces are trustworthy. For example, medial OFC activity reflected an increasing function of trustworthiness for self-resembling faces (Platek et al., 2009), and of personality traits estimated from sentences describing hypothetical actions (Tsukiura and Cabeza, 2011b). Taken together, activity of the medial OFC region could be modulated by facial attractiveness and positive attributes of personality estimated from faces.

The important role of the medial OFC in the processing of rewards has been demonstrated by cognitive neuroscience studies involving animal and human subjects (Rolls, 2000; Martin-Soelch et al., 2001; McClure et al., 2004; O'Doherty, 2004). For example, single unit recording studies with non-human primates have shown that the medial OFC contributes to the coding of reward values of stimuli (Rolls et al., 1989; Critchley and Rolls, 1996). Likewise, functional neuroimaging studies involving human subjects have reported that activity in the medial OFC region is associated with coding rewards from a variety of sensory modalities, including taste, olfaction, somatosensory, auditory, and vision, as well as more abstract rewards such as money (O'Doherty, 2004). In addition, one study found greater activity in this region when subjects viewed beautiful paintings than when they viewed ugly paintings, regardless of the category of painting (Kawabata and Zeki, 2004). Thus, the response bias toward affectively positive signals from faces could be strongly associated with reward-related activity in the medial OFC region. This implication is supported by functional neuroimaging evidence, in which attractive faces increased activations in the medial OFC as well as the nucleus accumbens comprising the putative reward circuit (Cloutier et al., 2008).

However, several studies have implied that roles in the OFC region may be dissociable between medial and lateral portions of this region. For example, one fMRI study reported that activity in the medial OFC region was positively correlated with an increasing function of facial attractiveness, whereas the lateral OFC region showed a reverse pattern of activity (O'Doherty et al., 2003). Another fMRI study, which investigated activations associated with the onset or offset of emotional expressions, demonstrated that the medial OFC region showed greater activity during the processing of positive expressions (offset of angry expression), and that activity in the lateral OFC region reflected both conditions of negative (onset of angry expression and offset of happy expression) and positive expressions (offset of angry expression) (Muhlberger et al., 2011). These findings suggest that roles of the lateral OFC in the processing of face-based affective signals may be different from those of the medial OFC, but the precise roles in the lateral OFC region are still controversial.

## Roles of the insular cortex in the processing of face-based affective signals

**Figure 3:**
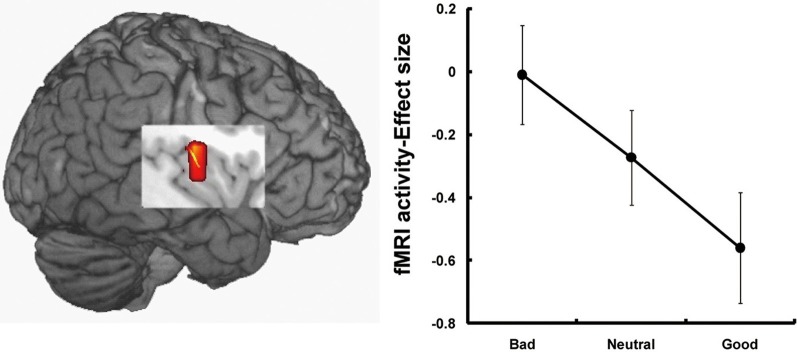
**Insular activations in response to untrustworthy faces (Tsukiura et al., 2012).** Activity of the insular cortex was greater during the processing of faces with an impression of untrustworthiness. This figure was reused with the permission.

The third candidate region associated with the processing of face-based affective signals is the insular cortex (Figure 3). Cognitive neuroscience studies have reported that the insular cortex is involved in the processing of affectively negative facial expressions, in particular expressions of disgust (Phillips et al., 1997, 1998; Sprengelmeyer et al., 1998; Sambataro et al., 2006). Additionally, insular activations during the processing of negative facial expressions have been identified in association with expressions of pain (Botvinick et al., 2005), or with the offset of happy expressions and the onset of angry expressions (Muhlberger et al., 2011). There is also functional neuroimaging evidence that the insula shows greater activity during the processing of unattractive faces than during that of attractive faces (O'Doherty et al., 2003; Krendl et al., 2006; Tsukiura and Cabeza, 2011b), and during the processing of untrustworthy faces than of trustworthy faces (Winston et al., 2002; Krendl et al., 2006; Tsukiura et al., 2012). Thus, insular activities could be modulated by face-based negative signals, which include both external features, such as negative facial expressions or unattractiveness of faces, and negative personality traits, such as untrustworthiness.

Functional neuroimaging studies have demonstrated that the insular cortex shows increasing activity associated with a variety of negative social situations, including social exclusion (Eisenberger et al., 2003), unfairness (Sanfey et al., 2003), and unreciprocated cooperation (Rilling et al., 2008). The insular activity has also been linked to the processing of pain (Critchley et al., 2000) and aversive conditioning (Seymour et al., 2004). These findings suggest that the insular cortex could contribute to an avoidance response away from people with face-based negative signals. The link between insular cortex and avoidance responses is consistent with findings that insular activity is involved in the anticipation of threat (Seymour et al., 2007), the avoidance of risky options in decision-making tests (Kuhnen and Knutson, 2005), and individual differences in avoidance learning (Samanez-Larkin et al., 2008).

## Roles of the hippocampus and fusiform gyrus in memory for faces

One of the important regions in memory for face-related information is the hippocampus. Functional neuroimaging studies have reported that the hippocampus as the medial temporal lobe (MTL) memory system is involved in the encoding and retrieval of episodic memory details including contextual information of an event (Davachi, 2006; Diana et al., 2007; Eichenbaum et al., 2007). The importance of this region have also been observed in the encoding and retrieval of face-related memories (Small et al., 2001; Sperling et al., 2001, 2003; Paller et al., 2003; Zeineh et al., 2003; Kirwan and Stark, 2004; Chua et al., 2007; Tsukiura et al., 2008, 2011). For example, one fMRI study showed hippocampal activations during the successful retrieval of both face-name associations and face-job title associations, and the hippocampal activations were significantly declined in older adults, compared to young adults (Tsukiura et al., 2011). In addition, hippocampal contributions to face-related memories have been identified in the processing of memory for face-scene associations (Hayes et al., 2010), or face-face and face-laugh associations (Holdstock et al., 2010). These findings suggest that the hippocampus could be involved in memory for details of face-related information.

Another important region in face memories is the right fusiform region, in which a region selective to the processing of face information is known as the fusiform face area (FFA; Kanwisher et al., 1997). Functional neuroimaging studies have reported that both FFA and hippocampal regions show significant activations during the successful encoding or retrieval of facial stimuli (Prince et al., 2009; Skinner et al., 2010). One theory of episodic memory consolidation proposes that components forming episodic memories are stored in unimodal or heteromodal association cortices, and that the hippocampus binds these components with event-specific contextual information (Alvarez and Squire, 1994; Mishkin et al., 1997; Nadel and Moscovitch, 1997; Fujii et al., 2000; Shastri, 2002; Norman and O'Reilly, 2003). Taken together, face memories could be successfully encoded or retrieved by the FFA-hippocampal network, in which facial stimuli processed in FFA are bound with the specific contextual information by the hippocampus.

## Neural mechanisms underlying the effects of face-based affective signals on memory for faces

**Figure 4:**
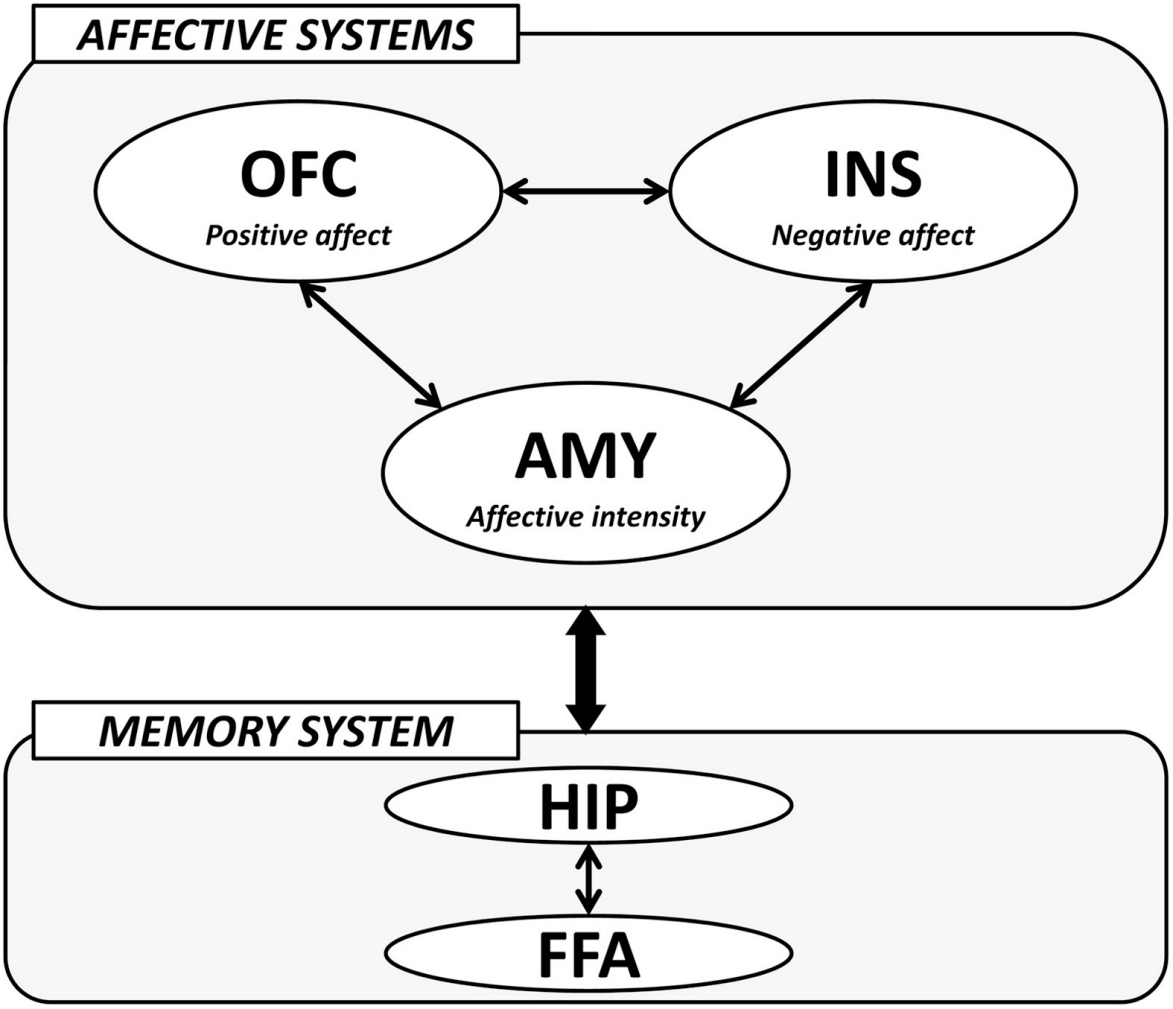
**A hypothetical model of the neural mechanisms underlying the effect of face-based affective signals on memory for faces.** AMY, amygdala; OFC, orbitofrontal cortex; INS, insular cortex; HIP, hippocampus; FFA, fusiform face area.

The importance of the amygdala, medial OFC, and insula in the processing of face-based affective signals has been identified in cognitive neuroscience studies. These studies have demonstrated that the amygdala contributes to the processing of both positive and negative affective signals from faces, and that medial OFC activity shows biased responses to positively affective signals from faces, whereas insular activity is modulated by negatively affective signals from faces. In addition, recent advances in effective connectivity analysis in functional neuroimaging studies have shown that the effective connectivity between the amygdala and medial OFC contributes to the differentiation of positive and neutral facial expressions from negatively valenced angry, disgust, and fear expressions (Liang et al., 2009), and that the effective connectivity between the amygdala and insula is associated with the processing of facial expressions of disgust (Tettamanti et al., 2012). Another fMRI study revealed that activities in the medial OFC region, which is involved in the processing of facial attractiveness and personality goodness, were negatively correlated with activities in the insular cortex, which is involved in the processing of facial unattractiveness and personality badness (Tsukiura and Cabeza, 2011b). These findings suggest that the amygdala-medial OFC-insula network could contribute to forming impressions of people by face-based affective signals. The amygdala could act as a primary system of face-based affective signals by responding to their intensity, and could adjust interacting activities between the medial OFC, which is involved in the processing of positively valenced signals from faces, and insula, which is involved in the processing of negatively valenced signals from faces (Figure 4).

The modulatory effects of face-based affective signals on memory for faces could be mediated by an interaction between *affective systems* including the amygdala, medial OFC, and insula, and *memory system* including the hippocampus and FFA (Figure 4). For example, functional neuroimaging studies have reported that both hippocampal and FFA regions show significant activations during the encoding and retrieval of faces (Prince et al., 2009; Skinner et al., 2010), and that faces with emotional expressions are remembered more accurately than those with neutral expressions by an effect of the affect-related amygdala (Fenker et al., 2005) or medial OFC (Tsukiura and Cabeza, 2008) activities on the memory-related hippocampal activities. The effects of face-based affective signals on memory for faces have also been identified in the processing of attractive faces, better memory for which is modulated by an interaction between activities in the medial OFC and hippocampus (Tsukiura and Cabeza, 2011a). Moreover, an interaction between the insula, which is associated with the processing of untrustworthy impressions, and the hippocampus is important in the enhancement of memory for untrustworthy faces (Tsukiura et al., 2012). These findings suggest that the amygdala, medial OFC, and insula as *affective systems* could directly or indirectly interact with the hippocampus-FFA network as *memory system*, and that the functional connection could contribute to the enhancement of memory for faces by face-based affective signals. Given that the interactions between affect-related regions and memory-related regions were identified in encoding (Tsukiura and Cabeza, 2011a; Tsukiura et al., 2012), retrieval (Fenker et al., 2005), or both (Tsukiura and Cabeza, 2008), the functional connection could be shared between these processes.

However, the possible interaction between regions as *affective systems* and *memory system* in memory for (affective) faces has been identified in memory for other affective stimuli such as affective pictures (LaBar and Cabeza, 2006; Dolcos et al., 2012). Thus, the model presented here is preliminary or tentative, because evidence whether an interaction between *affective*- and *memory*-related regions in our model is applied only to the memory for faces or to the affective memory in general is still scarce. Additional supports by future studies would be needed to clarify the neural mechanisms involved in the modulatory effects of face-based affective signals on memory for faces, and which of these mechanisms are specific to facial stimuli.

## Conclusion

This review article outlined the neural mechanisms underlying the effects of facial impressions on memory for faces by discussing previous functional neuroimaging findings. Information related to face-based affective signals consists of several factors such as facial expression, facial attractiveness, and trustworthiness, and is mediated by the amygdala, medial OFC, and insular regions as the affective system. Behaviorally, memory for faces is often enhanced by these kinds of face-based affective signals, and the memory enhancement is explained in terms of the modulatory effects of affect-related regions on the memory-related hippocampal region. In our daily lives, we have some impression of others on the basis of face-based affective signals, and the impression of others is important in deciding whether they should be approached or avoided. These effects of enhancement (approach) and impairment (avoidance) on perceiving people are influential in memory for faces. Face-based affective signals, as well as interpersonal relationship (Cacioppo and Cacioppo, 2012; Powers and Heatherton, 2012), could be important in remembering who should be approached or avoided in the context of social interaction.

### Conflict of interest statement

The author declares that the research was conducted in the absence of any commercial or financial relationships that could be construed as a potential conflict of interest.
